# Does Antioxidant Mitoquinone (MitoQ) Ameliorate Oxidative Stress in Frozen–Thawed Rooster Sperm?

**DOI:** 10.3390/ani12223181

**Published:** 2022-11-17

**Authors:** Lingwei Sun, Mengqian He, Jiehuan Xu, Caifeng Wu, Shushan Zhang, Defu Zhang, Jianjun Dai, Jun Gao

**Affiliations:** 1 Institute of Animal Science and Veterinary Medicine, Shanghai Academy of Agricultural Sciences, Shanghai 201106, China; 2 Division of Animal Genetic Engineering, Shanghai Municipal Key Laboratory of Agri-Genetics and Breeding, Shanghai 201106, China; 3 Key Laboratory of Livestock and Poultry Resources (Pig) Evaluation and Utilization, Ministry of Agriculture and Rural Affairs, Shanghai 201106, China

**Keywords:** mitoquinone (MitoQ), rooster semen, cryopreservation, antioxidant, apoptotic sperm

## Abstract

**Simple Summary:**

The cryopreservation of sperm cells is widely used in many animal species. In commercial stocks, frozen–thawed rooster semen is unreliable for artificial insemination. During cryopreservation, spermatozoa experience mitochondrial oxidative damage, causing oxidative damage to the spermatozoa. As a selective mitochondrial antioxidant, mitoquinone (MitoQ) has proven to be effective in ameliorating mitochondrial dysfunction and reducing oxidative stress in mitochondria. In this study, we attempted to determine whether MitoQ ameliorates oxidative stress in frozen–thawed rooster sperm, thereby improving sperm quality. We found that an adequate amount of MitoQ (150 nM) not only ameliorated post-thawed sperm quality and motility parameters by restoring ATP level and preventing membrane damage, but also improved the redox balance and antiapoptotic activity. These results can offer some new perspectives to improve the efficacy of the current rooster semen cryopreservation technology.

**Abstract:**

In this study, we aimed to determine the benefit of mitoquinone (MitoQ) in rooster semen extenders on sperm quality, motility parameters, antioxidant capacities, and apoptotic changes in post-thawed rooster semen. A total of 85 ejaculates from 18 roosters were collected and then divided into five equal aliquots and cryopreserved in extenders with 1.0% soy lecithin nanoparticles that contained various concentrations of MitoQ (0 nM (M0), 50 nM (M50), 100 nM (M100), 150 nM (M150), and 200 nM (M200)). By using a computer-assisted semen analyzer, sperm motility parameters were assessed after freeze thawing. The M150 group had significantly higher percentages of total motility, progressive motility, viability, acrosome membrane integrity, and mitochondrial activity than the other groups (*p* < 0.05). Compared to other groups, M100 and M150 groups produced a higher percentage of plasma membrane integrity and ATP contents (*p* < 0.05). Additionally, the lowest levels of ROS and MDA in spermatozoa were observed in M150 group (*p* < 0.05), whereas the highest levels of ROS and MDA were observed in sperm in the controls or the M200 group (*p* < 0.05). Significantly higher values of SOD, GPx, and Cas-3 were found in the M150 group compared to other groups (*p* < 0.05). Overall, these results demonstrate that MitoQ at 150 nM not only ameliorates post-thawed sperm quality and motility parameters by restoring ATP levels and preventing membrane damage, but also improves redox balance and antiapoptotic activities.

## 1. Introduction

The cryopreservation of sperm cells is widely used in many animal species. There are many benefits to cryopreservation including maintaining genetic diversity by increasing the diffusion and measurement of genetic progress and decreasing the number of males in the flock [1]. After decades of development, the technology for semen cryopreservation and artificial insemination (AI) is widely used in the dairy cattle industry, where optimization, standardization, and automation have been achieved in the cryopreservation of semen. In contrast, other livestock mammals have not yet achieved the same extent of success with respect to the cryopreservation of sperm such as poultry [2]. Although intensive research has been conducted for years, more work remains in order for poultry sperm to be cryopreserved successfully.

At the moment, a variety of freezing protocols involving different cryoprotectants and packaging methods are being implemented to minimize the freezing damage rate of frozen sperm for cryopreserving poultry semen. A previous study by our group demonstrated that the addition of 1.0% soy lecithin nanoparticles (nano-SL) to the rooster sperm extender significantly improved the quality of frozen/thawed semen and fertilization after artificial insemination in roosters [3]. However, semen cryopreservation remains in the laboratory stage and has not been used in commercial applications and genetic resource conservation in the poultry industry as yet. Frozen–thawed poultry sperms are of lower quality and result in poor fertilization rates because of their unique morphological and physiological features. The tails of poultry spermatozoa (80–90 µm) are longer than those of bull spermatozoa (50–60 µm). Long-tail sperm cells can be easily damaged during freezing or thawing [3]. There is not much difference in the diameter between the head and tail of a poultry sperm [4]. Consequently, sperm heads have smaller cytoplasmic volumes, which implies a lower movement ability in the cryoprotectants. During cryopreservation, the intracellular generation of reactive oxygen species (ROS) in the cryo-injured mitochondria may represent another crucial factor in decreasing sperm fertility, which results in oxidative damage to the spermatozoa [5]. 

In recent years, many studies have attempted to supplement mitochondrial-specific antioxidants for freezing extenders to protect spermatozoa against oxidative stress injuries [6]. Mitoquinone (MitoQ), a novel mitochondrial-targeted antioxidant, has proven to be effective in improving mitochondrial functions and attenuating oxidative stress in a mitochondrion [7]. Various studies have demonstrated that MitoQ is capable of preventing a number of disease-induced oxidative damage such as obese asthma, hypertension, and sepsis [8]. Limited studies on animals and humans have shown that adding MitoQ to the extenders improved the quality of post-thaw semen by decreasing the levels of oxidative stress. Fang et al. previously reported that MitoQ significantly improved the post-thaw viability of catfish sperm and reduced the damage produced by the enhancement in lipid peroxidation [9]. Similar observations have been reported in humans, showing that MitoQ protected the function and fertilization of thawed sperm [6]. However, the opposite effect was reported in bulls; semen extenders containing MitoQ did not improve sperm quality for cryopreservation [10]. As far as we know, no related studies on MitoQ have been reported in poultry semen cryopreservation. The objective of this study was to evaluate the influences of MitoQ in rooster semen extenders on the post-thaw quality of sperm.

## 2. Materials and Methods

### 2.1. Chemicals

Except where noted, all chemicals were supplied by Sigma-Aldrich (St. Louis, MO, USA).

### 2.2. Animals

A total of 18 Ross broiler breeder roosters (28 weeks old) were used for experiments at the Shanghai Academy of Agricultural Sciences, Shanghai, China. Each rooster was caged separately (size = 70 cm × 70 cm × 85 cm) with a 15/19 h light/dark cycle. The birds were fed the commercial feed of breeding flocks. Water was available at all times *ad libitum*. 

### 2.3. Semen Collection and Evaluation

Semen samples were collected twice a week during 3 weeks by the dorsoabdominal massage method [11]. A total of 96 ejaculates were obtained successfully. The obtained samples were then transferred into the semen collection tubes. For primary evaluation, samples were kept in a water bath at 35 °C for 5 min after collection. Prior to cryopreservation, only the ejaculates that met these criteria were subjected to subsequent analyses: a volume of ≥0.2 mL, semen concentration of ≥4.0 × 10^9^ spermatozoa/mL, a sperm motility value of ≥80%, and abnormal morphology of ≤10% [3]. From the 96 ejaculates, 11 ejaculates were unable to be used for subsequent analyses due to their inadequate quality. To reduce individual differences, semen samples after each collection were subsequently pooled and divided into five aliquots according to the experiment’s design.

### 2.4. Semen Processing and Sperm Cryopreservation

The extension of semen samples was performed with an SL nanoparticle-based extender consisting of 3.6342 g Tris, 1.8252 g citric acid, 0.5044 g glucose, and 6 mL glycerol in 100 mL of distilled water containing 2% SL nanoparticles. The concentration of SL nanoparticles was selected based on the beneficial effects reported by our previous research [3]. In the treatments, five extenders were supplemented with MitoQ (BioVision, Catalogue No. B1309-5) at various concentrations: 0 nM (M0, as control), 50 nM (M50), 100 nM (M100), 150 nM (M150), and 200 nM (M200). The pH and osmolality of the extenders were 7.05 and 340 mOsm/kg, respectively.

Semen cryopreservation was processed as described previously, with some modifications [12]. Briefly, semen samples were diluted to a final concentration of 50 × 10^6^ spermatozoa/mL with the extenders (1:20 *v*/*v*) at room temperature. Samples were cooled at 5 °C for 15 min and then loaded into 0.25 mL plastic straws. After 20 min of exposure to liquid nitrogen vapors, the straws were placed for at least one month in liquid nitrogen. The frozen straws were thawed in a 37 °C water bath for 20 s; then, various sperm functions and oxidative stress parameters were assessed.

### 2.5. Sperm Concentration and Morphology Assessment

Sperm concentrations were determined using a standard hemocytometer (HBG, Germany) and the results were expressed as 10^6^ spermatozoa/mL. Hancock solutions (21.4 mM formalin, 426 mM sodium, 304.29 mM Na_2_HPO_4_, and 99.42 mM K_2_HPO_4_) were used to assess the sperm’s morphology [13]. To detect abnormal acrosomes, approximately 200 spermatozoa were observed using phase contrast microscopy at 1000× magnification. 

### 2.6. Sperm Motility and Viability

Computer-Assisted Semen Analyzer (CASA) software was used to measure sperm motility. For each sample treatment, the semen sample (5 μL) was loaded into a pre-warmed 20 μm depth Leja-4 slide and placed on a microscopic stage at 37  °C. At least five fields with approximately 200 spermatozoa were analyzed for each sample.

The eosin–nigrosin method was used to assess the sperm viability [14]. Sperm suspension was diluted 1:1 in eosin–nigrosin stains. One drop of this mixture was smeared onto a glass slide and fully air dried. At least 200 spermatozoa in five microscopic fields were examined under a bright field at 400× magnification. The results are expressed as a percentage of live spermatozoa. 

### 2.7. Plasma Membrane and Acrosome Integrities

An assessment of the spermatozoa membrane integrity was conducted using the hypoosmotic swelling test (HOST) [15]. Briefly, a mixture of 100 µL semen samples and 1 mL HOST solution was incubated at 37 °C for 40 min. A drop of mixture was placed onto the pre-warmed microscope slide and then imaged with a phase contrast microscope. At least 200 spermatozoa in five microscopic fields were examined under a bright field at 400× magnification. Results are expressed as spermatozoa with swollen tails (%).

### 2.8. Acrosome Integrity

A fluorescein isothiocyanate–peanut agglutinin (FITC-PNA) test was conducted on the sperm acrosome membrane [16]. Mixing was performed with 500 µL of the semen sample, 0.5 µL of PNA-FITC (1.25 mg/mL final concentration), and 5 µL of propidium iodide (PI, 12 µM final concentration). After 15 min of incubation in the dark, the mixture was rinsed twice with PBS. At least 200 spermatozoa in five microscopic fields were evaluated under an epifluorescence phase-contrast microscope at 400× magnification. Spermatozoa with green staining over the acrosomal cap had an intact acrosome.

### 2.9. Mitochondrial Activity

A combination of Rhodamine 123 (R123) and PI was used to measure the sperm mitochondrial activities [17]. Concise mixing was performed with 25 µL of sperm suspension (1.5 × 10^6^ spermatozoa/mL), 10 µL of Rh123 (0.5 mM), and 10 µL of PI (300 mM). After 30 min of incubation at 37 °C in the dark, the mixture was evaluated under an epifluorescence phase-contrast microscope at 400× magnification. At least 200 spermatozoa in five microscopic fields were evaluated. A green fluorescence signal was detected in the midpiece of spermatozoa to confirm the presence of active mitochondria.

### 2.10. Adenosine Triphosphate (ATP) Concentration

In accordance with the manufacturer’s instructions, the concentration of sperm ATP was measured with an ATP Assay Kit (Beyotime Institute of Biotechnology, Shanghai, China). First, ATP lysis was applied to 1 mL aliquots containing 50 × 10^6^ sperm on ice. The mixture was then centrifuged at 12,000× g for 5 min at 4 °C. For the assay, the supernatant was collected and used. By using an ATP assay lysate, the ATP standard solution (0.5 mM) was diluted to concentrations of 10 nM to 10 M. Then, 40 µL of supernatant was mixed with 100 µL of an ATP-detection working dilution in 96-well plates. The absorbance was measured using a spectrophotometer at 636 nm. ATP concentrations were calculated using a standard curve for each sample.

### 2.11. ROS Level

ROS production was detected using dihydroethidium (DHE) fluorescence intensity, as previously described [17]. Samples were centrifuged at 700× *g* for 10 min and resuspended with 1 mL of PBS. From each sample, 100 µL (1.0 × 10^6^ spermatozoa/mL) was probed for 20 min at 37 °C with 5 µM DHE in the dark. The absorbance was measured using a spectrophotometer at 570 nm. Increased ROS generation correlates directly with increased fluorescence intensity. The ROS levels were represented as arbitrary units.

### 2.12. Superoxide Dismutase (SOD) Activity

The activity of sperm SOD was measured with a SOD Assay Kit (Jiancheng Biological Institute, Nanjing, China). Assays were conducted using the xanthine oxidase method based on the generation of superoxide anion (O_2_^−^). An equal volume of phosphate buffered sodium (50 mM, pH 7.0) was added to each sample at a ratio of 1:5. A total of 1 mL sample was added to a test tube and mixed with 1 mL of assay solution (sodium carbonate buffer (50 mM), xanthine (0.1 mM), nitroblue tetrazolium (0.025 mM), ethylenediaminetetraacetic acid (0.1 mM), and xanthine oxidase (0.1 units)) [18]. A spectrophotometric measurement of SOD activity at a wavelength of 525 nm was conducted and expressed as U/mL.

### 2.13. Malondialdehiyde (MDA) Concentration

By using the thiobarbituric acid reaction (TBA), the MDA in semen was determined as a measure of lipid peroxidation [12]. As a measure of TBA substance quantity, the absorption curve was compared with the standard curve for MDA equivalent generated by the acid catalyzed hydrolysis of 1, 1, 3, 3-tetramethoxypropan. To obtain the protein precipitate, the post-thaw semen sample (250 × 10^6^ spermatozoa/mL) was diluted 1:1 in cold 20% TBA. The mixture was collected via a centrifuge at 1000× *g* for 10 min. Then, the obtained supernatant (1 mL) was incubated at 100 °C with 0.67% TBA (1 mL) for 10 min and then cooled with tap water. With a spectrophotometer, the absorbance was measured at 532 nm. The results are expressed in nM.

### 2.14. Glutathione Peroxidase (GPx) Activity

The method used to measure the GPx activity has been previously described [19]. The reaction substance was composed as follows: potassium phosphate buffer (pH 7.0, 50 mM), sodium azide (1 mM), EDTA (1 mM), decreased nicotinamide adenine dinucleotide phosphate (0.2 mM), glutathione reductase (1 enzyme unit), and glutathione (1 mM). Prior to the start of the reaction, the sample (0.1 mL) was blended with a reaction substance (0.8 mL), incubated at 25 °C for 5 min, and 0.1 mL of 0.25 mM peroxide solution was added to induce the reaction. With a spectrophotometer, the absorbance was measured at 412 nm. The results are expressed in U/L.

### 2.15. Caspase 3 (Cas-3) Activity

In the internal pathway of apoptosis, Cas-3 is involved in the cascade of caspase activation. According to the manufacturer’s instructions, the Cas-3 activity of the sperm from each of the thawed samples was determined using a colorimetric kit (ab39401, Abcam). The MDA test’s extracted protein was utilized to gauge Cas-3 activity. The final concentration of each sample was adjusted to 1 mg/mL. Each sample was diluted 1:1 in a 2× reaction buffer containing 10 mM DTT and 5 μL of the DEVD-p-NA substrate (4 mM). After 60 min of reaction at 37 °C, microplate readers were used to measure absorbance at 405 nm. A duplicate measurement was performed on each sample.

### 2.16. Statistical Analysis

An analysis of the data was performed using SPSS 19.0 (SPSS, Chicago, IL, USA). Statistical data are expressed as mean ± standard deviation (SEM). Data from different groups were compared using one-way ANOVA followed by Tukey’s post hoc test. A difference was considered significant if the *p*-value was less than 0.05.

## 3. Results

A total of 85 ejaculates were collected from 18 roosters. According to the results, the means of the sperm volume, sperm concentration, sperm motility, sperm viability, and sperm malformation rate were as follows: 0.32 ± 0.06 mL, (4.77 ± 0.50) × 10^9^ spermatozoa/mL, 81.50 ± 3.18%, 89.62 ± 2.70%, and 6.36 ± 0.58%, respectively.

Table 1 shows the effect of MitoQ on post-thawed sperm motility parameters in rooster semen. The percentages of total motility (MOT) and progressive motility (PMOT) were significantly higher in M150 than that of M0, M50, M100, and M200 (*p* < 0.05). In comparison with the other groups, M150 showed significantly improved linearity (LIN) and beat cross frequency (BCF) (*p* < 0.05). The M100 and M150 groups had a significantly higher percentage of sperm STR compared to the other treated groups (*p* < 0.05). The other motility parameters (average path velocity (VAP), straight line velocity (VSL), curvilinear velocity (VCL), and the amplitude of the lateral head displacement (ALH)) did not differ significantly between groups (*p* > 0.05). 

Table 2 shows the effect of MitoQ treatments on the post-thawed sperm parameters of rooster semen. A significantly higher percentage of viability value was observed in the M150 group compared to the other groups (*p* < 0.05), except for the M100 group. The M100 group did not differ significantly from those obtained for the other groups (*p* > 0.05). The percentages of the sperm membrane’s integrity significantly increased in the M150 group compared to the other MitoQ concentration groups and the control groups (*p* < 0.05). The percentages of acrosome and mitochondrial activities were significantly higher in semen treated with the M150 group than those in the other MitoQ concentration groups and the control groups (*p* < 0.05). In addition, the M100 and M150 groups showed the highest levels of ATP compared to the M0, M50, and M200 groups (*p* < 0.05). 

According to the findings given in Table 3, the levels of sperm ROS and MDA were significantly lower in the M150 group compared to the other groups (*p* < 0.05), whereas the greatest levels were observed in the controls and/or the M200 group (*p* < 0.05). The highest significant SOD was observed in the M150 group compared to the other groups (*p* < 0.05). Higher values of GPx were observed in the M150 group compared to the other MitoQ concentration groups and the control groups (*p* < 0.05), but they did not differ between the control and the M50, M100, and M200 groups (*p* > 0.05). A significant increase in Cas-3 activity was observed in the control group compared to the treatment groups (*p* < 0.05), but the lowest significant Cas-3 activity was observed in the M150 group compared to the other groups (*p* < 0.05). 

## 4. Discussion

Few details have been provided about the protective role of MitoQ in the extender for rooster semen cryopreservation. This study not only helped us understand the influence of MitoQ on the sperm motion parameters and quality, but also enabled us in assessing the changes in enzymatic antioxidant activities.

The CASA, as an effective screening technique, has been proven to be successful for assessing sperm functions in multiple species [20]. First, we evaluated the sperm MOT and PMOT, which is a prerequisite for the successful fertilization of spermatozoa in vivo [21]. Frozen–thawed rooster semen with MitoQ concentrations of 150 nM showed a significant increase in MOT and PMOT and decreased with a higher concentration (200 nM). Similar observations of the antioxidant lycopene have also been previously reported in rooster sperm cryopreservation [22]. This may be due to excessive antioxidants leading to numerous negative consequences [23]. Despite no significant changes in the sperm kinematics parameters (VAP, VSL, VCL, and ALH) in the supplemented groups compared to the controls, the highest values of BCF, STR, and LIN were found at a concentration of 150 nM. In individual sperm, BCF is approximately proportional to the beating flagellar frequency. It is possible that fertilizing sperm needs sufficient BCF to traverse the female reproductive tract to complete fertilization [24]. This also explains the importance of BCF as the important indicator to measure the ability of sperm to fertilize. Moreover, other sperm motion characteristic parameters (STR and LIN) not only had positive correlations with litter sizes in pigs [25], but also with fertility in humans [26].

In general, the damage caused by sperm cryoinjury includes structural damage and functional change such as the sperm’s plasma membrane and acrosome. Spermatozoa plasma membrane integrity is highly related to the ability of sperms to fertilize [27]. Results from this study illustrated that MitoQ concentrations ranging between 50 and 150 nM had a protective effect for membrane integrity; however, 200 nM of MitoQ interrupts the membrane’s integrity. This finding is also comparable to another study in human spermatozoa that demonstrated that MitoQ at 200 nM could interrupt the membrane’s integrity, which resulted in mitochondrial dysfunction [6]. Moreover, mitochondrial dysfunction generally leads to a reduction in ATP production by impairing oxidative phosphorylation and the citric acid cycle [28]. A murine study found that MitoQ ameliorated mitochondrial dysfunction in mutant Htt neurons by increasing the levels of mitochondrial ATP [29]. The present study further demonstrates that a suitable concentration for the addition of MitoQ (between 50 and 150 nM) can improve the mitochondrial damage caused by cryopreservation. Furthermore, the acrosomal integrity of spermatozoa is critical for the sperm’s fertilization ability, which is closely associated with capacitation, a normal acrosome reaction [30]. The present study showed that positive effects of MitoQ were observed in the acrosome’s integrity with respect to thawed sperm. Another study confirmed that coenzyme Q10 (CoQ10, the active component of MitoQ) contributes to stabilizing the acrosome membrane of rooster semen during cryopreservation [31]. 

Maintaining a healthy biological system requires balancing oxidation and antioxidation. Notably, antioxidants are reported to have pro-oxidant effects, which are associated with high doses [32]. The data presented show that concentration ranges that are between 50 and 150 nM (MitoQ) result in antioxidant effects compared to high concentrations (200 nM). In tests on yellow catfish sperm, MitoQ was, however, not toxic at 200 nM [9]. Similarly to our studies, a treatment study on breast cancer cell lines reported that a sign of toxicity was observed with MitoQ at 113 nM [33]. Different types of cells or species responded remarkably differently to MitoQ. The reason for this discrepancy may be due to a substantial variation existing in the spermatozoa’s membrane composition in terms of cholesterol and saturated and unsaturated phospholipids. It has been demonstrated that exogenous antioxidants with MitoQ can provide effective treatments for combating the excessive production of ROS during freezing or thawing processes when its concentration is high enough in the mitochondria’s inner membrane [6]. We found that increasing the concentrations of MitoQ was toxic to the spermatozoa. The reason for this may be that excess MitoQ can stimulate the release of one-electron oxidizing species such as O_2_^•−^. There have been reports of excessive levels of oxidative stress induced by O_2_^•−^ [34]. In addition, the O_2_^•−^ anion radical is a key determinant of the overall effects of ROS [35]. It is possible to speculate that, for the above reason, we achieved high levels of ROS in a 200 nM concentration added to the extender, as described in this study. 

It has been suggested that cryopreservation could decrease the mitochondrial membrane potential and the membrane’s permeability, subsequently inducing apoptosis in spermatozoa [36]. MitoQ has been shown to reduce mitochondrial ROS, and specifically, it has been shown to introduce antiapoptotic effects [37]. The caspase family plays a central role in apoptosis, particularly Cas-3. As an apoptotic effector gene, Cas-3 activation can induce cell apoptosis, and the expression of Cas-3 is positively correlated with the rate of apoptosis in cells [38]. Thus, the level of Cas-3 was measured to determine the effect of MitoQ on sperm apoptosis. Our findings support the above contention that MitoQ supports sperm freezing by playing a protective anti-apoptosis role, as lower levels of Cas-3 were detected after the MitoQ treatments.

## 5. Conclusions

In conclusion, the findings of the present study indicated that suitable and additional concentrations of MitoQ (150 nM) in the freezing extender of rooster sperm cryopreservation not only ameliorated post-thaw sperm quality and motility parameters by restoring ATP levels and preventing membrane damage but also improved redox balance and antiapoptotic activities. The results presented here provide an excellent starting point for the further analysis of the mechanism of action of MitoQ and to assess the effect of MitoQ on the ability of sperm to fertilize.

## Figures and Tables

**Table 1:** Influence of different concentrations of mitoquinone (MitoQ) on the motility parameters of rooster sperm assessed by CASA.

Items (Unit)	Concentrations ^1^				
	M0 (Controls)	M50 (50 nM)	M100 (100 nM)	M150 (150 nM)	M200 (200 nM)
MOT (%)	52.56 ± 1.42 ^b^	51.80 ± 2.77 ^b^	58.14 ± 1.95 ^b^	62.93 ± 1.26 ^a^	45.01 ± 3.09 ^c^
PMOT (%)	45.28 ± 2.81 ^b^	42.18 ± 1.06 ^c^	50.39 ± 2.63 ^b^	56.71 ± 0.98 ^a^	38.11 ± 2.99 ^c^
VAP (µm/s)	90.17 ± 1.88	88.47 ± 2.36	91.47 ± 0.85	90.63 ± 2.52	89.78 ± 2.36
VSL (µm/s)	32.12 ± 2.41	30.16 ± 3.10	40.87 ± 2.35	44.15 ± 2.31	43.02 ± 2.19
VCL (µm/s)	77.61 ± 6.28	74.75 ± 7.17	86.40 ± 8.70	85.11 ± 5.96	76.55 ± 7.18
ALH (µm)	6.15 ± 1.28	6.22 ± 0.87	7.55 ± 1.18	7.92 ± 0.63	6.11 ± 1.16
BCF (Hz)	22.16 ± 3.16 ^c^	25.70 ± 2.52 ^c^	30.83 ± 1.09 ^b^	34.95 ± 1.18 ^a^	28.57 ± 4.06 ^c^
STR (%)	34.47 ± 2.63 ^b^	35.34 ± 1.98 ^b^	44.28 ± 2.16 ^a^	48.46 ± 3.69 ^a^	46.13 ± 1.17 ^a^
ALIN (%)	43.67 ± 1.82 ^c^	41.66 ± 1.89 ^c^	46.32 ± 1.05 ^b^	54.49 ± 2.15 ^a^	50.56 ± 2.06 ^b^

^1^ A different superscript indicates a significant difference among groups within a row. Abbreviations: total motility (MOT), progressive motility (PMOT), average path velocity (VAP), straight line velocity (VSL), curvilinear velocity (VCL), amplitude of the lateral head displacement (ALH), beat cross frequency (BCF), straightness (STR), and linearity (LIN).

**Table 2:** Influence of different concentrations of mitoquinone (MitoQ) on the post-thawed sperm quality parameters of rooster semen.

Items (Unit)	Concentrations ^1^				
	M0	M50	M100	M150	M200
Viability (%)	44.26 ± 4.15 ^b^	50.57 ± 3.66 ^b^	54.14 ± 2.47 ^ab^	59.21 ± 2.78 ^a^	51.58 ± 1.82 ^b^
Membrane integrity (%)	39.94 ± 3.70 ^c^	47.17 ± 2.08 ^b^	52.02 ± 3.95 ^a^	56.11 ± 2.18 ^a^	46.23 ± 3.06 ^b^
Acrosome integrity (%)	39.49 ± 1.57 ^c^	47.19 ± 1.99 ^b^	49.46 ± 1.83 ^b^	54.10 ± 1.84 ^a^	45.86 ± 2.67 ^b^
Mitochondrial activity (%)	44.14 ± 1.20 ^c^	45.38 ± 2.31 ^b^	52.56 ± 1.46 ^b^	57.97 ± 1.26 ^a^	47.11 ± 2.50 ^c^
ATP (nM/10^7^ spermatozoa)	2.55 ± 0.24 ^c^	2.68 ± 0.15 ^c^	3.50 ± 0.44 ^a^	3.38 ± 0.32 ^a^	3.11 ± 0.2 ^ab^

^1^ A different superscript indicates a significant difference among groups within a row. Abbreviations: adenosine triphosphate (ATP).

**Table 3:** Influence of different concentrations of mitoquinone (MitoQ) on the antioxidant capacities of post-thawed rooster semen.

Items (Unit)	Concentrations ^1^				
	M0 (Controls)	M50 (50 nM)	M100 (100 nM)	M150 (150 nM)	M200 (200 nM)
ROS	0.75 ± 0.05 ^b^	0.70 ± 0.10 ^b^	0.69 ± 0.04 ^b^	0.64 ± 0.06 ^c^	0.78 ± 0.07 ^a^
SOD (U/mL)	133.74 ± 16.40 ^d^	178.96 ± 5.13 ^c^	190.33 ± 4.39 ^b^	216.25 ± 10.33 ^a^	146.89 ± 10.78 ^d^
MDA (mM)	8.89 ± 0.59 ^a^	8.10 ± 0.25 ^a^	7.46 ± 0.13 ^b^	6.86 ± 0.28 ^c^	7.90 ± 0.41 ^a^
GPx (U/L)	98.28 ± 10.25 ^b^	106.74 ± 8.40 ^b^	115.94 ± 5.30 ^b^	128.62 ± 6.44 ^a^	110.33 ± 7.63 ^b^
Cas-3 (OD. 450 nm)	0.16 ± 0.02 ^a^	0.12 ± 0.02 ^b^	0.09 ± 0.01 ^b^	0.06 ± 0.01 ^c^	0.08 ± 0.01 ^b^

^1^ A different superscript indicates a significant difference among groups within a row. Abbreviations: reactive oxygen species (ROS), superoxide dismutase (SOD), malondialdehyde concentration (MDA), glutathione peroxidase (GPx), and caspase 3 (Cas-3).

## Data Availability

Not applicable.

## References

[1] Santiago-Moreno J., Blesbois E. (2020). Functional aspects of seminal plasma in bird reproduction. Int. J. Mol. Sci..

[2] ÇİFTCİ H.B., AYGÜN A. (2018). Poultry semen cryopreservation technologies. Worlds Poult. Sci. J..

[3] Sun L.W., He M.Q., Wu C.F., Zhang S.S., Dai J.J., Zhang D.F. (2021). Beneficial influence of soybean lecithin nanoparticles on rooster frozen-thawed semen quality and fertility. Animals.

[4] Thurston R.J., Hess R.A. (1987). Ultrastructure of spermatozoa from domesticated birds: Comparative study of turkey, chicken and guinea fowl. Scanning Microsc..

[5] Mehdipour M., Kia H.D., Martínez-Pastor F. (2020). Poloxamer 188 exerts a cryoprotective effect on rooster sperm and allows decreasing glycerol concentration in the freezing extender. Poult. Sci..

[6] Kumar P., Wang M., Isachenko E., Rahimi G., Mallmann P., Wang W., von Brandenstein M., Isachenko V. (2021). Unraveling subcellular and ultrastructural changes during vitrification of human spermatozoa: Effect of a mitochondria-targeted antioxidant and a permeable cryoprotectant. Front. Cell Dev. Biol..

[7] Smith R.A., Murphy M.P. (2010). Animal and human studies with the mitochondria-targeted antioxidant mitoq. Ann. N. Y. Acad. Sci..

[8] Piscianz E., Tesser A., Rimondi E., Melloni E., Marcuzzi A. (2021). Mitoq is able to modulate apoptosis and inflammation. Int. J. Mol. Sci..

[9] Fang L., Bai C.L., Chen Y.H., Dai J., Xiang Y., Ji X.P., Huang C.J., Dong Q.X. (2014). Inhibition of ROS production through mitochondria-targeted antioxidant and mitochondrial uncoupling increases post-thaw sperm viability in yellow catfish. Cryobiology.

[10] Cmara D.R., Ibanescu I., Siuda M., Bollwein H. (2022). Mitoquinone does not improve sperm cryo-resistance in bulls. Reprod. Domest. Anim..

[11] Burrows W.H., Quinn J.P. (1937). The collection of spermatozoa from the domestic fowl and turkey. Poult. Sci..

[12] Ye N., Lv Z., Dai H., Huang Z., Shi F. (2021). Dietary alpha-lipoic acid supplementation improves spermatogenesis and semen quality via antioxidant and anti-apoptotic effects in aged breeder roosters. Theriogenology.

[13] Masoudi R., Sharafi M., Shahneh A.Z. (2019). Effects of coq10 on the quality of ram sperm during cryopreservation in plant and animal based extenders. Anim. Reprod. Sci..

[14] Appiah M.O., Li W., Zhao J., Liu H., Lu W. (2020). Quercetin supplemented casein-based extender improves the post-thaw quality of rooster semen. Cryobiology.

[15] Appiah M.O., He B.B., Lu W.F., Wang J. (2019). Antioxidative effect of melatonin on cryopreserved chicken semen. Cryobiology.

[16] Thananurak P., Chuaychu-Noo N., Thélie A., Phasuk Y., Vongpralub T., Blesbois E. (2020). Different concentrations of cysteamine, ergothioneine, and serine modulate quality and fertilizing ability of cryopreserved chicken sperm—Sciencedirect. Poult. Sci..

[17] Salih S.A., Kia H.D., Mehdipour M., Najafi A. (2021). Does ergothioneine and thawing temperatures improve rooster semen post-thawed quality?. Poult. Sci..

[18] Sowińska M., Paukszto U., Jastrzbski J.P., Bukowska J., Ciereszko A. (2020). Transcriptome analysis of turkey (meleagris gallopavo) reproductive tract revealed key pathways regulating spermatogenesis and post-testicular sperm maturation. Poult. Sci..

[19] Najafi D., Taheri R.A., Najafi A., Shamsollahi M., Alvarez-Rodriguez M. (2020). Effect of astaxanthin nanoparticles in protecting the post-thawing quality of rooster sperm challenged by cadmium administration. Poult. Sci..

[20] Holt W.V., O"Brien J., Abaigar T. (2007). Applications and interpretation of computer-assisted sperm analyses and sperm sorting methods in assisted breeding and comparative research. Reprod. Fertil. Dev..

[21] El-Khawagah A., Kandiel M., Samir H. (2020). Effect of quercetin supplementation in extender on sperm kinematics, extracellular enzymes release, and oxidative stress of egyptian buffalo bulls frozen–thawed semen. Front. Vet. Sci..

[22] Najafi A., Taheri R.A., Mehdipour M., Farnoosh G., Martínez-Pastor F. (2018). Lycopene-loaded nanoliposomes improve the performance of a modified beltsville extender broiler breeder roosters. Anim. Reprod. Sci..

[23] Vaithiyanathan V., Mirunalini S. (2015). Assessment of antioxidant potential and acute toxicity studies of whole plant extract of pergularia daemia (forsk). Toxicol. Int..

[24] Winters R.A., Hamilton D.N., Bhatnagar A.S., Fitzgerald R., Bovin N., Miller D.J. (2018). Porcine sperm binding to oviduct cells and glycans as supplements to traditional laboratory semen analysis. J. Anim. Sci..

[25] Holt C., Holt W.V., Moore H., Reed H., Curnock R.M. (1997). Objectively measured boar sperm motility parameters correlate with the outcomes of on-farm inseminations: Results of two fertility trials. J. Androl..

[26] Larsen L., Scheike T., Jensen T.K., Bonde J.P., Ernst E., Hjollund N.H., Zhou Y., Skakkebaek N.E., Giwercman A. (2000). Computer-assisted semen analysis parameters as predictors for fertility of men from the general population. The danish first pregnancy planner study team. Hum. Reprod..

[27] Noh S., Go A., Kim D.B., Park M., Jeon H.W., Kim B. (2020). Role of antioxidant natural products in management of infertility: A review of their medicinal potential. Antioxidants.

[28] Wiland E., Fraczek M., Olszewska M., Kurpisz M. (2016). Topology of chromosome centromeres in human sperm nuclei with high levels of DNA damage. Sci. Rep..

[29] Yin X.L., Manczak M., Reddy P.H. (2016). Mitochondria-targeted molecules mitoq and ss31 reduce mutant huntingtin-induced mitochondrial toxicity and synaptic damage in huntington’s disease. Hum. Mol. Genet..

[30] Kumar N., Lone S.A., Prasad J.K., Jan M.H., Ghosh S.K. (2016). Effect of egg yolk powder on freezability of murrah buffalo (bubalus bubalis) semen. Vet. World..

[31] Masoudi R., Sharafi M., Shahneh A.Z., Kohram H., Nejati-Amiri E., Karimi H., Khodaei-Motlagh M., Shahverdi A. (2018). Supplementation of extender with coenzyme q10 improves the function and fertility potential of rooster spermatozoa after cryopreservation. Anim. Reprod. Sci..

[32] Bouayed J., Bohn T. (2010). Exogenous antioxidants--double-edged swords in cellular redox state: Health beneficial effects at physiologic doses versus deleterious effects at high doses. Oxid. Med. Cell Longev..

[33] Rao V.A., Klein S.R., Bonar S.J., Zielonka J., Shacter E. (2010). The antioxidant transcription factor Nrf2 negatively regulates autophagy and growth arrest induced by the anticancer redox agent mitoquinone. J. Biol. Chem..

[34] Suzuki C., Hatayama N., Ogawa T., Nanizawa E., Otsuka S., Hata K., Seno H., Naito M., Hirai S. (2020). Cardioprotection via metabolism for rat heart preservation using the high-pressure gaseous mixture of carbon monoxide and oxygen. Int. J. Mol. Sci..

[35] Li X.C., Chen B., Xie H., He Y.H., Zhong D.W., Chen D.F. (2018). Antioxidant structure–activity relationship analysis of five dihydrochalcones. Molecules.

[36] Taheri Moghadam M., Asadi Fard Y., Saki G., Nikbakht R. (2019). Effect of vitamin d on apoptotic marker, reactive oxygen species and human sperm parameters during the process of cryopreservation. Iran. J. Basic.Med. Sci..

[37] Hu Q.Y., Ren J.N., Li G.W., Wu J., Wu X.W., Wang G.F., Gu G.S., Ren H.J., Hong Z.W., Li J.S. (2018). The mitochondrially targeted antioxidant MitoQ protects the intestinal barrier by ameliorating mitochondrial DNA damage via the Nrf2/ARE signaling pathway. Cell Death Dis..

[38] Cheng Z., Xu H., Wang X., Liu Z. (2017). Lactobacillus raises in vitro anticancer effect of geniposide in HSC-3 human oral squamous cell carcinoma cells. Exp. Ther. Med..

